# Further Observations on the Treatment of Summer Complaint

**Published:** 1902-04

**Authors:** C. C. Cronkhite

**Affiliations:** Marion, Ind.


### FURTHER OBSERVATIONS ON THE TREATMENT OF SUMMER COMPLAINT.

By C. C. CRONKHITE, M.D.,

Marion, Ind.

Last year I reported some notes on an epidemic of dysentery which prevailed several years ago in Marion. Among the 23 cases recorded only two terminated fatally, and I believe that even these would have recovered with careful nursing. Owing to the severity of this epidemic the results obtained were unusually favorable, and are attributable in great part to the treatment adopted. In these cases it is very important to control the exhausting mucous and bloody discharges from the bowel, and for this purpose it is necessary to select an astringent which will exert an effect on that portion of the intestinal tract which is affected by the disease. The majority of astringents are unsuitable for this purpose, owing to the fact that they are absorbed or rendered inert in the upper portion of the intestine, so that the amount that finds its way into the lower portion is insufficient to produce any curative action. Moreover, it is very important in these cases not to administer remedies which may disturb the digestion, and this is very likely to happen with the astringents in common use. The attempt has been made in a number of the new astringent preparations introduced in recent years to overcome this objectionable feature. Of these, I employed tannigen with great success in the above epidemic. Its administration was unattended with the least gastric irritation, this being attributable to the fact that the drug is insoluble in the gastric juice. In the intestinal canal, however, the drug gradually yields up its tannic acid constituent, owing to the action of the alkaline fluids, and this liberation of tannic acid is particularly marked at the points where the secretion is most abundant; that is to say, at the site of the disease.

Since reporting my observations with tannigen I have had an opportunity of making a further study of its properties during last summer, and would cite a number of cases from among those treated, in order to throw further light upon its mode of action :

CASE I. Babe, 14 month old ; frequent, large, watery and offensive stools, which condition had existed for thirty-six hours. I gave tannigen in three grain doses every hour until four doses were taken ; then at intervals of four hours ; also teaspoonful doses of elixir of pepsin with food, and ordered rectal douches night and morning. Recovery after one week.

CASE II. Mrs. S., 25 years of age ; vomiting and purging for four days, for which she had been taking home remedies. I prescribed a brisk cathartic and followed with ten grain doses of tannigen every two hours, until the actions of the bowels were checked, and then gave it every four to six and eight hours as needed. Recovery after three days.

CASE III. Babe, four months old, had been under the care of a doctor for a week. Frequent, watery and musty stools, green and full of undigested milk. Nursing at the breast discontinued for twelve hours; sub-chloride of mercury was given in minute doses hourly for six hours, followed by tannigen, 2 grain doses, every two hours, until the movements were checked, then every four hours. Disappearance of the diarrhea after seven days.

CASE IV. Girl, 2 years of age, had suffered for twenty-four hours. Vomiting and purging from eating a large quantity of green corn. I again gave calomel followed by ten grain doses of tannigen. The vomiting ceased after three hours, and the diarrhea after forty-four hours.

CASE V. Babe, 2 months old, had been allowed green apples, which caused diarrhea. Yellowish green mucous and fetid stools hourly. I ordered the rectal douche night and morning, and tannigen three grains every two hours, and later four to six hours. The father reported on the following morning that the child was greatly improved. Recovery in four days.

CASE VI. Girl, 12 months old ; diarrhea of two days' duration ; ten to twelve whitish-yellow, offensive and copious stools in in twenty-four hours. Treatment commenced by triturates of calomel every hour till the evacuated matter assumed a more normal consistency and color; then tannigen in three grain doses, every three hours until its effect was produced, and then every four hours. Recovery complete in ten days.

CASE VII. Girl, 6 months old, bottle-fed. Diarrhea for four days; copious green, watery and offensive stools, occurring every one or two hours during the day and night. Loss of appetite and sleeplessness, and great thirst and vomiting. All food was stopped for twelve hours, and the bowels moved thoroughly with calomel; this was followed by my usual remedy—tannigen. In this case I was compelled to prescribe a small amount of opiates to relieve the pain, but all the time continued the use of tannigen. This child has not fully recovered and is in a precarious condition.

CASE VIII. G., 40 years of age; ate an unusually hearty supper, and awoke the next morning with severe vomiting and a diarrhea that kept him "on the jump all the time." I gave him 15-grain doses of tannigen every hour until the action of the bowels was checked, then at intervals of four or five hours. I also used in this case half grain tablets of opium and camphor to relieve pain. Patient out in two days.

CASE. IX. Mr. H., 39 years of age, teamster; was seen by me at 9 a. m. The bowels had moved ten times during the previous night. Intense thirst and nausea and severe abdominal pain were present. This case was treated similarly to the above. Recovery occurred in two days.

These are a few of the many cases of diarrhea treated during the past year with tannigen. The reader will observe that I gave the drug alone, and I have obtained better results by so doing. I have tried the various formulas with tannigen, but was not pleased with the results.

Tannigen is almost devoid of taste and children take it readily. Its action is quick, powerful and effective. When called to a case at the commencement of diarrhea no preliminary treatment is needed, but I begin at once with tannigen, giving small, oft-repeated doses, rather than large ones. Where cases have received "home treatment" for several days, a calomel purge is an excellent preliminary step, and this is to be followed with tannigen. To the doctor who has been in the habit of treating diarrhea with castor oil and other nauseous drugs, tannigen presents the advantages of being well tolerated by the child, and thus pleasing the parents. I have not had a death so far this year from summer complaint. It is of the utmost importance to restrict the diet and prevent the child from receiving too much fluids. There is a desire on the part of the parents to do this, and they must be closely watched by the doctor.

